# LINC00662 Promotes Aggressive Traits by Modulating OCT4 Expression through miR-335-5p in Gallbladder Cancer Cells

**DOI:** 10.3390/ijms25126740

**Published:** 2024-06-19

**Authors:** Pablo Pérez-Moreno, Ismael Riquelme, Carolina Bizama, Luis Vergara-Gómez, Julio C. Tapia, Priscilla Brebi, Patricia García, Juan Carlos Roa

**Affiliations:** 1 Programa de Comunicación Celular en Cáncer, Facultad de Medicina Clínica Alemana, Universidad del Desarrollo, Santiago 7780272, Chile; p.perez@udd.cl; 2 Institute of Biomedical Sciences, Faculty of Health Sciences, Universidad Autónoma de Chile, Temuco 4810101, Chile; ismael.riquelme@uautonoma.cl; 3 Millenium Institute on Immunology and Immunotherapy (IMII), Centro de Prevención y Control de Cancer (CECAN), Department of Pathology, School of Medicine, Pontificia Universidad Católica de Chile, Santiago 8380000, Chile; cbizamas@uc.cl (C.B.); pgarciam@uc.cl (P.G.); 4 Centre of Excellence in Translational Medicine (CEMT), Scientific and Technological Bioresource Nucleus (BIOREN), Biomedicine and Translational Research Lab, Universidad de la Frontera, Temuco 4810296, Chile; luisosvaldo.vergara@ufrontera.cl; 5 Programa de Biología Celular y Molecular, Instituto de Ciencias Biomédicas, Facultad de Medicina, Universidad de Chile, Santiago 8380453, Chile; jtapiapineda@uchile.cl; 6 Laboratory of Integrative Biology (LIBi), Millennium Institute on Immunology and Immunotherapy (MIII), Center for Excellence in Translational Medicine—Scientific and Technological Bioresource Nucleus (CEMT-BIOREN), Universidad de La Frontera, Temuco 4810296, Chile; priscilla.brebi@ufrontera.cl

**Keywords:** LINC00662, gallbladder cancer, miR-335-5p, OCT4

## Abstract

Long non-coding RNAs (lncRNAs) are nucleotide sequences that participate in different biological processes and are associated with different pathologies, including cancer. Long intergenic non-protein-coding RNA 662 (LINC00662) has been reported to be involved in different cancers, including colorectal, prostate, and breast cancer. However, its role in gallbladder cancer has not yet been described. In this article, we hypothesize that LINC00662 has an important role in the acquisition of aggressiveness traits such as a stem-like phenotype, invasion, and chemoresistance in gallbladder cancer. Here, we show that LINC00662 is associated with larger tumor size and lymph node metastasis in patients with gallbladder cancer. Furthermore, we show that the overexpression of LINC00662 promotes an increase in CD133^+^/CD44^+^ cell populations and the expression of stemness-associated genes. LINC00662 promotes greater invasive capacity and the expression of genes associated with epithelial–mesenchymal transition. In addition, the expression of LINC00662 promotes resistance to cisplatin and 5-fluorouracil, associated with increased expression of chemoresistance-related ATP-binding cassette (ABC) transporters in gallbladder cancer (GBC) cell lines. Finally, we show that the mechanism by which LINC00662 exerts its function is through a decrease in microRNA 335-5p (miR-335-5p) and an increase in octamer-binding transcription factor 4 (OCT4) in GBC cells. Thus, our data allow us to propose LINC00662 as a biomarker of poor prognosis and a potential therapeutic target for patients with GBC.

## 1. Introduction

Gallbladder cancer (GBC) is a highly lethal neoplasm that represents the most common cancer of the biliary tract, causing 89,055 deaths per year with an incidence of 1.2 cases per 100,000 individuals around the world [1]. Generally, GBC is diagnosed as an incidental finding in patients with cholelithiasis who are treated with surgery (cholecystectomy); unfortunately, in most cases, this finding occurs in advanced stages of the disease, causing a short life expectancy [2].

Most research in cancer disease has focused on protein-coding genes. However, in recent years non-coding RNAs (ncRNAs), such as microRNAs (miRNAs), small nuclear RNAs (snRNAs), PIWI-interacting RNAs (piRNAs), and long non-coding RNAs (lncRNAs), have acquired a relevant role in cancer, being implicated in different malignant processes such as invasion, stemness, and drug resistance [3].

LncRNAs are defined as RNA sequences that do not possess functional open reading frames (ORFs) and participate in different forms as signal molecules, guides, decoys, and/or competitive endogenous RNA networks (ceRNAs) [4,5]. The ceRNA mechanism has gained great relevance in regulating gene expression and modulating an aggressive phenotype in cancer cells [6]. For instance, lncRNA MINCR promotes invasion, proliferation, and tumorigenicity in GBC cells [7], and CCAT1, which is overexpressed in GBC tissues, and its expression is positively correlated with tumor status, lymph node invasion, and tumor–node–metastasis (TNM) stage via sponging miRNA-218-5p [8]. Interestingly, only the lncRNA downregulated in liver cancer stem cells (DILC) has been associated with stemness features in GBC cells promoting cancer stem cell (CSC)-like expansion and OCT4 gene expression [9].

An interesting lncRNA to study in GBC is long intergenic non-protein-coding RNA 662 (LINC00662), which has been assessed in other types of neoplasms, such as lung, breast, and prostate cancer. In addition, it has been previously associated with tumor progression, metastasis, and tumorigenesis, becoming a promising new biomarker for the prognosis and diagnosis of other cancers [10]. However, the role of LINC00662 in GBC is still unknown.

Thus, this article aims to elucidate the role and probable mechanism of LINC00662 in the acquisition of aggressive traits in GBC cells and whether its expression is associated with clinicopathological features in patients with GBC.

## 2. Results

### 2.1. LINC00662 Is Overexpressed in GBC Tissues and Shows an Uneven Expression in GBC Cells

**Figure 1:**
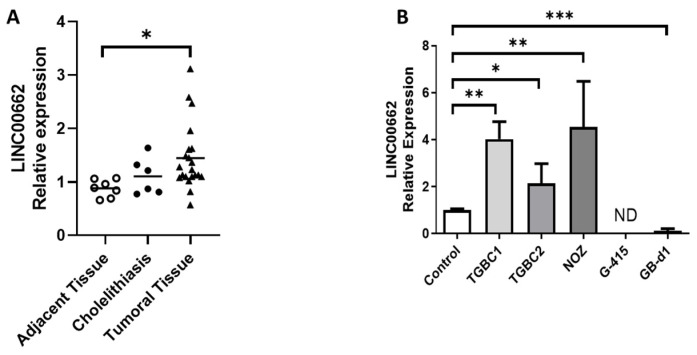
LINC00662 levels in gallbladder tissues and GBC cell lines. The total RNA obtained from each condition was converted into cDNA to perform qPCR for measuring LINC00662 levels. (**A**) Significantly higher LINC00662 expression was observed in GBC tissues than in adjacent and cholelithiasis tissues. Cholelithiasis did not show statistical differences between the groups. (**B**) GBC cell lines TGBC2, NOZ, and TGBC1 showed higher LINC00662 levels compared to control; meanwhile, GB-d1 cells showed lower LINC00662 expression, and in G-415 cells LINC00662 expression was not detected. The mean expression of 4 cholelithiasis tissue samples was used as a control. Data represent average ± SD (n = 3). ANOVA and the Tukey post-test were used. * *p* ≤ 0.05, ** *p* ≤ 0.01, *** *p* ≤ 0.001. ND: not detected.

RT-qPCR was performed in GBC, cholelithiasis, and non-tumoral adjacent tissue samples, as well as five different GBC cell lines. The data showed that LINC00662 expression was significantly elevated in the cancer tissues compared to the adjacent tissues (*p* = 0.0483); however, no significant differences were observed between the tumoral and cholelithiasis tissues (*p* = 0.3461), or between the cholelithiasis and adjacent tissues (*p* = 0.7259) (Figure 1A). The TGBC2, NOZ, and TGBC1 cell lines showed higher levels of LINC00662 than the control (the mean expression of four cholelithiasis tissue samples was used as a control). Interestingly, the TGBC1 cell line derived from lymph node metastasis expressed a 2-fold increase in LINC00662 compared with the primary tumor derived from the same patient (TGBC2 cell line). Conversely, G-415 cells showed no amplification of LINC00662, while GB-d1 cells showed low expression compared to the control (Figure 1B). Based on these results, G-415 and GB-d1 GBC cell lines were selected to perform overexpression of LINC00662 in the next experiments due to their low levels of LINC00662.

### 2.2. Association between LINC00662 Expression and Clinicopathological Features and GBC

The age percentage in each group of patients with gallbladder cancer was 61.5% greater than 63 years and 38.5% less than 63 years. Regarding gender, 26.6% were male and 73.4% were female. Tumor size was greater than 4.5 cm^2^ in 62.5% of the cases and less than 4.5 cm^2^ in 37.5% of the cases. Positive lymph node metastasis was present in 42.8% of the patients, while 57.2% had negative lymph node metastasis. Additionally, 71.4% of the patients were in stage III or IV of the disease, and 28.6% were in stage I or II. Regarding histological grade, 8.3% of the patients had grade 1, 66.7% had grade 2, and 25% had grade 3. Regarding clinicopathological information, LINC00662 expression was significantly increased in tumors larger than 4.5 cm^2^ (*p* = 0.049) (the value 4.5 cm^2^ was determined as the mean of the total tumor sizes) and in tumors with the presence of lymph node metastasis compared to those without lymph node metastasis (*p* = 0.028). However, we did not find statistical differences in the TNM stages of the disease (*p* = 0.186), gender (*p* = 0.825), age (*p* = 0.832), or grade (differentiated (Grade 1) vs. moderately differentiated (Grade 2) *(p* = 0.768); moderately differentiated (Grade 2) vs. undifferentiated (Grade 3) *(p* = 0.867); differentiated (Grade 1) vs. undifferentiated (Grade 3) (*p* = 0.937)). These data suggest that the expression of LINC0062 could be a biomarker of tumor growth and/or early biomarker of metastasis in patients with gallbladder cancer. A summary of the data is presented in Table 1.

### 2.3. LINC00662 Promotes CSC-like Features in GBC Cells

**Figure 2:**
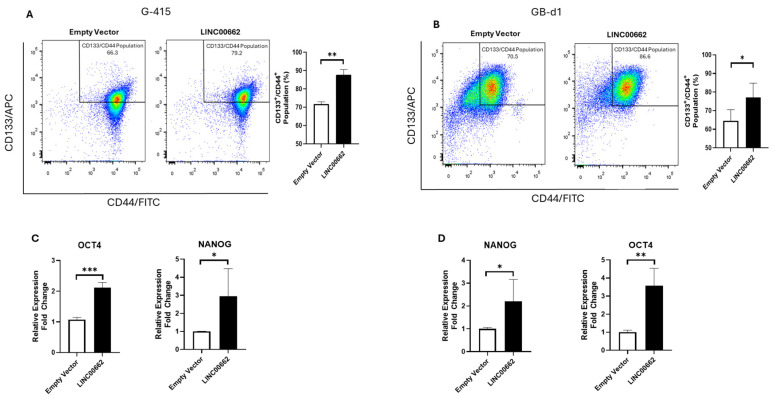
LINC00662 expression promotes CSC-like features in GBC cell lines. Overexpression of LINC00662 in (**A**) G-415 and (**B**) GB-d1 GBC cells increased CD133^+^/CD44^+^ population. (**C**) G-415 and (**D**) GB-d1 cells showed higher gene expression of NANOG and OCT4 after LINC00662 overexpression. Data represent average ± SD (n = 3). The *t*-test was used. * *p* ≤ 0.05, ** *p* ≤ 0.01, *** *p* ≤ 0.001.

The stem-like phenotype in cancer cells has been associated with aggressive features, such as resistance to chemotherapy, invasive/metastatic behavior, and tumorigenic capacity [11]. In addition, the stem-like phenotype is related to the overexpression of transcriptional factors such as homeobox protein nanog (NANOG) and octamer-binding transcription factor 4 (OCT4) as well as the expression of membrane proteins such as CD133 and CD44 [12]. Therefore, we evaluated whether overexpression of LINC00662 could promote higher CD133^+^/CD44^+^ cell populations in G-415 and GB-d1 cells by flow cytometry. We found that the overexpression of LINC00662 increased CD133^+^/CD44^+^ populations in both GBC cell lines (Figure 2A,B) and promoted a higher expression of genes associated with the CSC-like phenotype such as NANOG and OCT4 (Figure 2C,D). These results suggest that the expression of LINC00662 can enhance the expression of stemness-related genes and drive the generation of CSC-like features in vitro.

### 2.4. LINC00662 Promotes Drug Resistance in GBC Cell Lines

**Figure 3:**
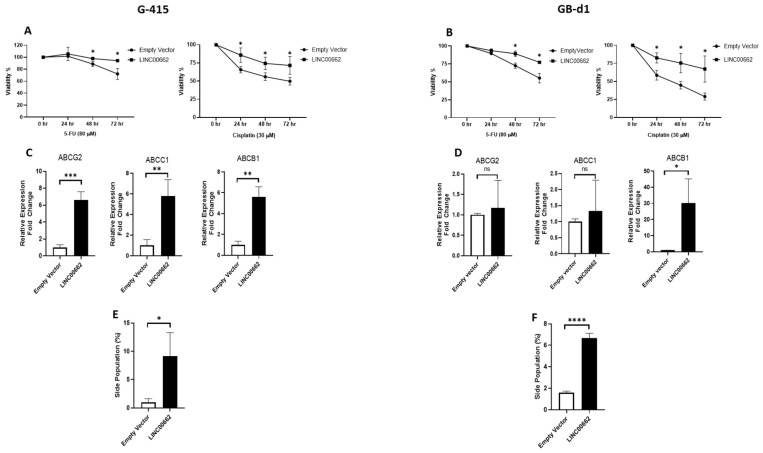
LINC00662 increased drug resistance in GBC cell lines. (**A**) G-415 and (**B**) GB-d1 cells showed higher cell viability under cisplatin (30 µM) and 5-FU (80 µM) treatments after LINC00662 overexpression compared to the empty vector. (**C**) G-415 cells with LINC00662 overexpression increased the mRNA expression of transporters ABCG2, ABCC1, and ABCB1; and (**D**) GB-d1 cells with LINC00662 overexpression increased the mRNA expression only of the transporter ABCB1. (**E**) G-415 and (**F**) GB-d1 cells increased the percentage of cells capable of pumping out DCV (side-population assay) after LINC00662 transduction. Data represent average ± SD (n = 3). The *t*-test was used. * *p* ≤ 0.05, ** *p* ≤ 0.01, *** *p* ≤ 0.001, *p* ≤ 0.0001, ****. ns: no significance.

To determine the role of LINC00662 in drug resistance, we treated G-415 and GB-d1 GBC cell lines with 5-fluorouracil (5-FU) and cisplatin. First, we generated a concentration curve for both chemotherapeutics to determine the optimal concentration. We determined that the optimal concentration was 80 µM for 5-FU and 30 µm for cisplatin because the cell viability was close to the IC50 (Figure S1). The results of the 3-(4,5-dimethylthiazol-2-yl)-5-(3-carboxymethoxyphenyl)-2-(4-sulfophenyl)-2H-tetrazolium (MTS) assays showed that the G-415 cell lines had greater resistance to 5-FU after 48 h and cisplatin after 24 h. The GB-d1 cell lines showed resistance to 5-FU after 48 h and cisplatin after 24 h (Figure 3A,B). Then, we evaluated whether this drug resistance was related to an increased expression of ATP-binding cassette (ABC) transporters, as suggested in the literature [13]. Interestingly, LINC00662 overexpression increased the mRNA levels of the transporters ABCG2, ABCC1, and ABCB1 in the G-415 cell line (Figure 3C). However, the GB-d1 cell line showed a significant increase only for the ABCB1 gene, without important differences for the ABCG2 and ABCC1 genes (Figure 3D). Finally, to correlate the potential drug resistance with the ability of ABC pumps to efflux antineoplastic drugs out of the cell, a side-population assay was performed using flow cytometry. This assay evaluates the activity of ABC transporters by expelling dye cycle violet (DCV) in the presence or absence of verapamil, an inhibitor of these pumps. As expected, overexpression of LINC00662 increased the percentage of cells capable of pumping out DCV in the G-415 (Figure 3E) and GB-d1 (Figure 3F) cell lines. These results indicate that LINC00662 can induce chemoresistance in GBC cell lines by increasing the expression and activity of ABC transporters.

### 2.5. LINC00662 Promotes Epithelial–Mesenchymal Transition (EMT)-Associated Invasiveness in GBC Lines

**Figure 4:**
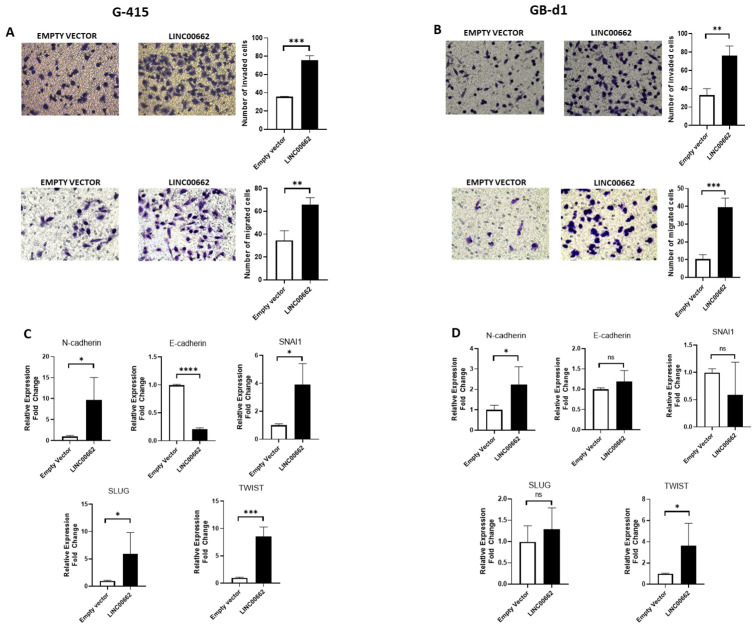
(**A**) G-415 and (**B**) GB-d1 cells with LINC00662 overexpression showed an increased invasion and migration capacity. (**C**) G-415 and (**D**) GB-d1 cells with LINC00662 overexpression were assessed by qPCR regarding the expression of EMT markers including N-cadherin, E-cadherin, SNAI1, SLUG, and TWIST. Data represent average ± SD (n = 3). The *t*-test was used. * *p* ≤ 0.05, ** *p* ≤ 0.01, *** *p* ≤ 0.001, **** *p* ≤ 0.0001. ns: no significance.

The invasive features of tumor cells are crucial to degrade and invade the extracellular matrix (ECM) to induce metastasis in other organs in the body [14]. The migration assay using a Boyden chamber and the invasion assay using the Boyden chamber with Matrigel matrix showed that LINC00662 overexpression promotes greater migration and invasion capacity in G-415 and GB-d1 cell lines (Figure 4A,B). In addition, LINC00662 overexpression also increased the mRNA levels of N-cadherin, SNAI1, SLUG, and TWIST, and decreased E-cadherin in the G-415 cells (Figure 4C). However, the GB-d1 cells evidenced a significant expression increase only for N-cadherin and TWIST (Figure 4D). Finally, overexpression of LINC00662 promotes a greater proliferation capacity at 72 h (Figure S2). These data suggest that LINC00662 promotes EMT features, consequently increasing the migration and invasive capacity in GBC cells.

### 2.6. Aggressive Traits in GBC Cells Are Induced by LINC00662/miR-335-5p/OCT4 Axis

**Figure 5:**
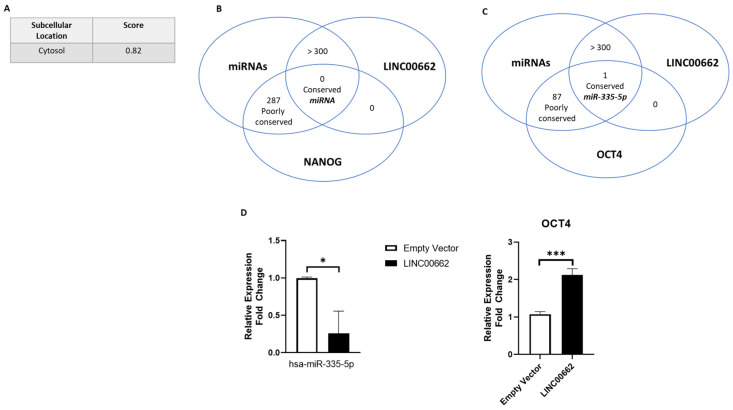
The LINC00662/miR-335-5p/OCT4 axis in GBC. (**A**) Localization score of LINC00662 shows that this lncRNA is predominantly located in the cytosol of the cell. (**B**,**C**) Venn diagram showing in silico analyses of interaction among LINC00662, miR-335-5p, and OCT4. (**D**) G-415 cells showed a decreased expression of miR-335-5p and an increased expression of OCT4 after LINC00662 overexpression. Data represent average ± SD (n = 3). The *t*-test was used. * *p* ≤ 0.05, *** *p* ≤ 0.001.

To determine the possible mechanism by which LINC00662 exerts its function, various in silico analyses were performed to determine (1) the localization of LINC00662 within the cell, and (2) the relationship between LINC00662 and miRNAs associated with stemness-inducing genes. The intracellular localization of LINC00662 was determined predominantly within the cytosol, with a score of 0.82 (http://lin-group.cn/server/iLoc-LncRNA(2.0)/pre.php (accessed on 11 March 2024)) (Figure 5A). Then, to determine which miRNAs had binding sites for NANOG or OCT4, a website tool was used (www.targetscan.com (accessed on 26 March 2023)), which found that miR-335-5p had conserved union sites for OCT4 but not NANOG (Figure 5B,C). Interestingly, miR-335-5p has been previously described as a tumor suppressor miRNA [15].

Later, an additional analysis performed with the LncBook 2.0 site (available at http://ngdc.cncb.ac.cn/lncbook/omics/interaction (accessed on 26 March 2023)) showed that the LINC00662 sequence has binding sites for miR-335-5p (Figure 5C), which strongly suggests that LINC00662 could bind miR-335-5p and decrease its expression in cancer. This idea was reaffirmed when the levels of miR-335-5p were shown to be decreased and levels of OCT4 were increased after the LINC00662 overexpression in the G-415 GBC cells (Figure 5D). Curiously, in the GB-d1 cells, miR-335-5p expression was not detected in either group (empty vector and LINC00662). These data suggest that overexpression of LINC00662 causes an almost 80% decrease in miR-335-5p expression levels and 2-fold increase in OCT4 expression levels that leads to the acquisition of characteristics associated with greater aggressiveness in GBC.

## 3. Discussion

In recent years, several studies have assessed the role of lncRNAs in tumor progression and have suggested them as diagnostic and prognostic biomarkers in cancer [16,17]. Specifically, LINC00662 has been described as an important oncogene in different types of neoplasms [10], being involved in proliferation and colony formation in lung cancer [18], or inducing poor prognosis in prostate cancer [19]. However, its role in GBC has not yet been determined. Our results showed that LINC00662 expression was higher in GBC tissues than in non-tumoral adjacent tissues and cholelithiasis tissues, even evidencing a significant positive correlation with tumor size and the presence of lymph node metastasis, which is similar to the results obtained in studies in lung, breast, and prostate cancers [18,19,20]. Interestingly, the levels of LINC00662 were slightly higher in cholelithiasis tissues than adjacent tissue, suggesting that LINC00662 could be a potential marker of tumor progression in patients with GBC due to cholelithiasis tissues possibly having preneoplastic alterations and/or acquired genetic alterations such as *TP53* and *FHIT* mutations and/or COX-2, TNF-α, and CLDN-18 overexpression, which have been observed to be precursors of cancer [21]. However, due to the small number of tissue samples, it is not possible to confirm completely this idea.

Our results have also shown that LINC00662 overexpression promotes some stem-like traits in GBC cells, including an increase in CD133^+^/CD44^+^ cell populations and the expression of stemness-associated genes such as *OCT4* and *NANOG*, which is strongly associated with greater aggressiveness in cancer cells [11,22,23]. These findings were similar to those found in breast cancer cell lines, where LINC00662 knockdown decreased the expression of OCT4 and NANOG, which decreased invasion, proliferation, and tumor growth, suggesting that this lncRNA could actively participate in the acquisition of the stem-like phenotype in breast cancer [24]. Another characteristic closely attributable to lncRNAs is the development of chemoresistance [25,26]. To date, the role of LINC00662 in chemoresistance has not been well determined, and even less so the effect of LINC00662 on cisplatin and 5-FU resistance in GBC. Our results have shown not only that LINC00662 promotes resistance to cisplatin and 5-FU but also increases the expression of ABC transporters, which are known to be closely associated with the increase in drug resistance of cancer stem cells [13]. In addition, this is the first evidence that indicates that LINC00662 promotes a greater activity of ABC transporters, which was confirmed through the side-population assay. Interestingly, the greater abundance of side-population cells has been closely related to a stem-like phenotype, which is a sign of higher aggressiveness in cancer [27,28].

**Figure 6:**
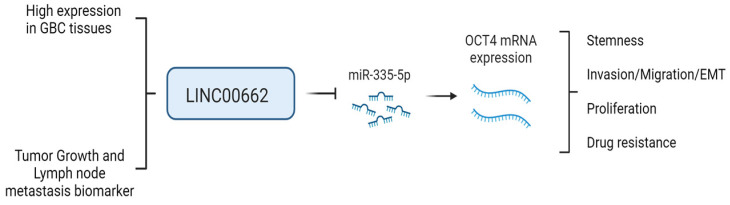
Representative scheme of the action of LINC00662 in GBC cells. Overexpression of LINC00662 induces downregulation of mIR-335-5p, allowing upregulation of OCT4. This promotes the acquisition of a stem-like phenotype, greater migration and invasive capacity, epithelial–mesenchymal transition, proliferation, and chemoresistance in gallbladder cancer cells.

The capability to invade other tissues and generate metastatic focus in the body is another aggressive trait [29]. Our data showed that LINC00662 overexpression was able to induce greater migration and invasiveness in GBC cells and promote the expression of markers associated with the EMT process. In this regard, previous studies demonstrated that LINC00662 promotes greater invasive capacity in lung and prostate cancer [19,30]. However, its relationship with EMT features has been poorly studied. For instance, a single previous study indicated that overexpression of LINC00662 was able to enhance invasion with higher vimentin and lower E-cadherin levels in oral squamous cell carcinoma [31]. These previous results were similar to ours, in which LINC00662 overexpression also produced increased N-cadherin and decreased E-cadherin levels, along with a higher expression of SNAI1, SLUG, and TWIST, which are considered as EMT driver genes and are also closely involved in the development of the stem-like phenotype [32,33]. Moreover, our data reveal significant differences in proliferation at 72 h between LINC00662 and the empty vector in both cell lines. Notably, it has been demonstrated that the CSC-like phenotype exhibits elevated proliferation rates, indicative of its robust tumor-forming potential in cancer [34]. For instance, the overexpression of the lncRNA PVT1-214 has been demonstrated to elevate proliferative capacity in colorectal cancer (CRC) cell lines, which is associated with traits linked to a stem-like phenotype such as enhanced spheroid formation, tumorigenesis, and metastasis in vivo [35]. Finally, we determined that miR-335-5p has a conserved site to bind OCT4 mRNA, which suggests that miR-335-5p may regulate OCT4 expression. This conclusion is widely supported by numerous articles that demonstrated a direct interaction between miR-335-5p and OCT4 acting as sponging [36,37,38,39]. Interestingly, our in silico analyses showed that LINC00662 has two binding sites for miR-335-5p that could induce the repression of this miRNA, which was then confirmed in G-415 cells in which LINC00662 overexpression decreased miR-335-5p expression. Regarding this, similar studies showed that LINC00662 knockdown decreases the levels of OCT4 binding to miR-144-3p in breast cancer cells [24]. Since there were not significant differences in miR-335-5p levels between the control and LINC00662-transduced groups in GB-d1 cells, it is suggested that the oncogenic effects observed by LINC00662 overexpression could be through a different mechanism of regulation, which is very common among lncRNAs due to the large number of probable targets [40]. Furthermore, G-415 and GB-d1 cells are cell lines extracted from different patients (information in http://cellosaurus.org/CVCL_8198 and http://cellosaurus.org/CVCL_H705, accessed on 12 March 2024), which could explain the cellular differences between them. In summary, this is the first article that shows the effect of the LINC00662/miR-335-5p/OCT4 axis in GBC cells, by which LINC00662 inhibits miR-335-5p, increasing OCT4 levels that finally promote the acquisition of more aggressive features in this malignancy (Figure 6).

## 4. Materials and Methods

**Figure 7:**
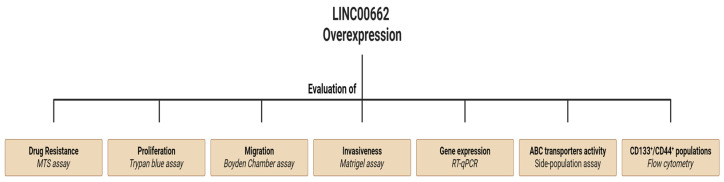
Scheme of general experimental design. LINC00662 was overexpressed into GBC G-415 and GB-d1 lines and drug resistance determined by viability MTS assay, invasive capacity by Matrigel-coated transwell assay, migration capacity by Boyden chamber assay, CD133^+^/44^+^ population by flow cytometry and proliferation by trypan blue assay. Expression of LINC00662, miR-335-5p, ABC transporters, stemness, and EMT-associated genes was evaluated by RT-qPCR. Activity of ABC transporters was evaluated by side-population assay.

General experimental design: LINC00662 was overexpressed in G-415 and GB-d1 GBC cell lines, and we evaluated the effect on the cellular properties of the stem-like phenotype, such as CD133^+^/44^+^ populations by flow cytometry and stemness-associated genes by RT-qPCR. Proliferation capacity by trypan blue assay. Invasion capacity was evaluated through Matrigel-coated transwell assays. Migration was evaluated by boyden chamber assay. EMT expression profile and miR-335-5p expression were determined by RT-qPCR. Chemoresistance was determined with a viability -(4,5-dimethylthiazol-2-yl)-5-(3-carboxymethoxyphenyl)-2-(4-sulfophenyl)-2H-tetrazolium (MTS) assay. ABC transporter expression and activity were determined by RT-qPCR and side-population assays, respectively. Finally, LINC00662 from adjacent, cholelithiasis, and tumoral tissues was measured with RT-qPCR (Figure 7).

### 4.1. Cell Culture and Sample Tissues

G-415 and GB-d1 cells were grown in RPMI-1640 medium (Thermo Fisher Scientific, Waltham, MA, USA) supplemented with 10% fetal bovine serum (FBS) and 1% penicillin/streptomycin (Thermo Fisher Scientific). NOZ, TGBC-1TKB, and TGBC-2TKB cells were grown in DMEM High Glucose (Corning Inc., Corning, NY, USA) supplemented with 5% FBS and 1% penicillin/streptomycin. A total of 34 fresh frozen tissues (GBC n = 21, cholelithiasis n = 6, and non-tumoral adjacent tissues n = 7) were obtained from the Biobank of Pontificia Universidad Católica de Chile, following the protocols to obtain written informed consent from each patient. The study protocol was approved by the Ethics Committee of Pontificia Universidad Católica de Chile CEC-Med UC 200911005.

### 4.2. Lentiviral Transduction

A full-length LINC00662 cDNA sequence was inserted into the multicloning region of plasmid pLVX-PURO (Clontech, Mountain View, CA, USA). Lenti-X 293 T cells (Clontech, USA) were transfected with Lenti-X packaging single shots (Clontech) and with 7 µg of lentiviral plasmid containing LINC00662 cDNA or an empty vector. At 48 h post-transfection, supernatants containing lentiviral particles were harvested and passed through a cellulose acetate filter with a pore size of 0.45 μm. Virus production was verified with Lenti-X GoStix Plus (Clontech). Finally, cell transduction was realized with 6 × 10^6^ cells/well in plates of 100 mm, with the recombinant lentiviruses at an MOI of 5 under normal growth conditions. At 24 h post-transduction, the cells were selected with puromycin (Invivogen, San Diego, CA, USA) 7.5 µg/mL for 72 h. Cells were examined with a Nikon Eclipse TS100 Inverted Microscope (Nikon, Melville, NY, USA). Figure S3 demonstrates that overexpression of LINC00662 was successfully achieved in both cell lines. In GB-d1 cells, LINC00662 expression increased 50-fold, while in G-415 cells, LINC00662 became detectable by qPCR.

### 4.3. RT-qPCR

Total RNA for mRNA quantification was extracted from cells using the EZNA Total RNA Kit I (Omega Bio-tek, Norcross, GA, USA) and treated with DNase I (Omega Bio-tek, Norcross, GA, USA). Total RNA for miRNA quantification was extracted from cells using the miRNeasy Micro Kit (Qiagen, Germantown, MD, USA). Reverse transcription for mRNAs was performed using the AffinityScript QPCR cDNA Synthesis Kit (Agilent Technologies, Santa Clara, CA, USA), according to the manufacturer’s directions. For miRNA reverse transcription, we used the miRCURY LNA RT Kit (Qiagen). Quantitative real-time PCR was performed in an AriaMX Real-time PCR system (Agilent Technologies) with SYBR Green PCR Master Mix (Agilent Technologies) for mRNAs and with miRCURY LNA miRNA PCR Assay (Qiagen) for miRNAs. Reactions were performed in triplicate, and the relative abundance of each RNA was determined by using the 2^−ΔΔCt^ method, using GAPDH for mRNAs and 5S rRNA for miRNAs as normalizing genes. Probes used were miR-335-5p Geneglobe ID: ZP00004680 and 5s rRNA Geneglobe ID: ZP00004680. The primers used were chosen using the online software Primer3 version 4.1.0 [41]. Primer sequences are described below in Table 2.

### 4.4. CD133^+^/44^+^ Populations by Flow Cytometry

For CD133^+^/CD44^+^ population analysis, 2 × 10^5^ cells were incubated with 5 μL (0.25 μg) 7-AAD (BioLegend, San Diego, CA, USA) as a viability marker and then with the surface marker antibodies anti-CD133/APC and anti-CD44/BV-421 (BioLegend). The proportion was 1 μL/1 × 10^5^ cells, diluted in 200 μL PBS/2% FBS, for 30 min. Unlabeled cells, APC mouse IgG1ƙ, and BV-421 mouse IgG1ƙ isotypes (BioLegend) were used as controls. Analyses were in a Becton-Dickinson LSRFortessa X-20 (Becton Dickinson, Franklin Lakes, NJ, USA) flow cytometer using FACSDIVA 8.02 software (Becton Dickinson, Franklin Lakes, NJ, USA) at the MED.UCHILE-FACS Facility (Facultad de Medicina, Universidad de Chile).

### 4.5. Side-Population Assay by Flow Cytometry

Cells (1 × 10^6^) were treated with 200 μM verapamil for 30 min (Sigma-Aldrich, St. Louis, MO, USA). Then, cells were incubated with 2 µL Vibrant DyeCycle violet Stain for 30 min (Invitrogen, Waltham, MA, USA), and finally washed and prepared for analysis in a Becton-Dickinson LSRFortessa X-20 (Becton Dickinson) flow cytometer. Analyses were performed using FACSDIVA 8.02 software (Becton Dickinson) at the MED.UCHILE-FACS Facility (Facultad de Medicina, Universidad de Chile).

### 4.6. Cell Migration

Cells (5 × 10^4^) were seeded in Boyden chambers placed in 24-well plates with 600 µL RPMI/10% FBS. Cells were incubated for 6 h at 37 °C 5% CO_2_. Boyden chambers were stained and fixed with 0.05% crystal violet in 20% methanol for 30 min. Migrated cells were counted under a Nikon E100 microscope (Nikon). Cells were counted in a total of 10 random fields.

### 4.7. Cell Invasion

Cells (5 × 10^4^) were seeded in Boyden chambers pre-coated with Cultrex Reduced Growth Factor Basement Membrane (#3433-010-R1, R&D Systems, Minneapolis, MN, USA) at a concentration of 40 µg and placed in 24-well plates with 600 µL RPMI/10% FBS. Cells were incubated for 16 h at 37 °C 5% CO_2_. Boyden chambers were stained and fixed with 0.05% crystal violet in 20% methanol for 30 min. Invasive cells were counted under a Nikon E100 microscope (Nikon). Cells were counted in a total of 10 random fields.

### 4.8. Viability Assay for Drug Resistance

Cells (1 × 10^4^) were seeded in 96-well plates and cultured in full growth medium overnight. Then, the medium was removed and replaced with 200 µL complete medium containing cisplatin 30 µM or 5-fluorouracil 80 µM. The plates were incubated at 37 °C, 5% CO_2_ for 72 h. After the treatment, cells were incubated with 20 µL of MTS reagent (CellTiter 96 AQueous One Solution Cell Proliferation Assay, Promega, Fitchburg, WI, USA) for 3 h at 37 °C. Then, the spectrophotometric absorbance of the samples was detected by using a Biotek Epoch plate reader (Agilent Technologies) at 490 nm.

### 4.9. Statistical Analysis

Data were first analyzed using the Shapiro–Wilk normality test. Then, for two groups the U Mann–Whitney test or *t*-test were used according to the detection in the normality test. For more than two groups, Kruskal–Wallis with Dunn post-test or ANOVA with Tukey post-test were used according to the detection in the normality test. Values were plotted as mean ± SD from at least three independent experiments. Statistical differences were determined by using the GraphPad Prism 5 software (GraphPad, Boston, MA, USA). A *p* value ≤ 0.05 was considered significant.

## 5. Conclusions

Overexpression of LINC00662 induces an increase in stem-like cell populations and stemness-related genes. In addition, it promotes aggressiveness traits such as greater invasive capacity and chemoresistance. This effect may occur through sponging miR-335-5p, allowing the increase in OCT4 levels. In addition, LINC00662 correlated with larger tumor size and lymph node metastasis, suggesting that LINC00662 could be a biomarker of tumor growth and early metastasis in patients with GBC. These findings suggest that LINC00662 could be a new therapeutic target in patients with GBC.

## Figures and Tables

**Table 1:** Relationship of LINC00662 expression with clinicopathological features in patients with gallbladder cancer.

Clinicopathological Features	% Patients	LINC00662 Expression (Mean ± SD)	*p* Value
Age			0.832
≥63	61.5	1.87 ± 1.47	
<63	38.5	1.62 ± 0.86	
Gender			
Male	26.6	1.94 ± 0.83	0.825
Female	73.4	1.4 ± 1.39	
Tumor size			
≥4.5 cm^2^	62.5	2.72 ± 1.47	0.049 *
<4.5 cm^2^	37.5	0.99 ± 0.16	
Lymph node metastasis			
Positive	42.8	2.36 ± 0.82	0.028 *
Negative	57.2	1.025 ± 0.15	
TNM stage			
I and II	28.6	1.025 ± 0.01	0.186
III and IV	71.4	2.36 ± 0.97	
Histological grade			
Grade 1	8.3	1.2 ± 0.14	
Grade 2	66.7	2.08 ± 1.43	
Grade 3	25	1.62 ± 0.76	
Grade 1 vs. Grade 2			0.768
Grade 2 vs. Grade 3			0.867
Grade 1 vs. Grade 3			0.937

* *p* ≤ 0.05. TNM: tumor–node–metastasis.

**Table 2:** Primers for RT-qPCR.

Gene	Forward Primer	Reverse Primer
*NANOG*	5′-AA CAAAGGATGAAGTGCAAGCGG	5′-TCCAAGTTGGGTTGGTCCAAGTCT
*OCT4*	5′-AAT TTG TTC CTG CAG TGC CC	5′-GCA GCC TCA AAA TCC TCT CG
*GAPDH*	5′-GAGTCAACGGATTTGGTCGT	5′-GACAAGCTTCCCGTTCTCAG
*ABCC1*	5′-CCG TGT ACT CCA ACG CTG ACA T	5′-ATG CTG TGC GTG ACC AAG ATC C
*ABCG2*	5′-GTT CTC AGC AGC TCT TCG GCT T	5′-TCC TCC AGA CAC ACC ACG GAT A
*ABCB1*	5′-GCT GTC AAG GAA GCC AAT GCC T	5′-TGC AAT GGC GAT CCT CTG CTT C
*N-cadherin*	5′-CCT CCA GAG TTT ACT GCC ATG AC	5′-GTA GGA TCT CCG CCA CTG ATT C
*E-cadherin*	Fw 5′-TGG AGG AAT TCT TGC TTT GC	5′-CGT ACA TGT CAG CCA GCT TC
*SNAI1*	5′-TGC CCT CAA GAT GCA CAT CCG A	5′-GGG ACA GGA GAA GGG CTT CTC
*TWIST*	5′-GCC AGG TAC ATC GAC TTC CTC T	5′-TCC ATC CTC CAG ACC GAG AAG G
*SLUG*	5′-ATC TGC GGC AAG GCG TTT TCC A	5′-GAG CCC TCA GAT TTG ACC TGT C
*LINC00662*	5′-GCT TCA TGA CTT GTG CCC AA	5′-CAC CAG TTT CAG AAG CGT GT

## Data Availability

The original contributions presented in the study are included in the article/Supplementary Material, further inquiries can be directed to the corresponding author.

## Supplementary Materials

The following supporting information can be downloaded at: https://www.mdpi.com/article/10.3390/ijms25126740/s1.
