# Supplementary material for: LINC00662 Promotes Aggressive Traits by Modulating OCT4 Expression through miR-335-5p in Gallbladder Cancer Cells
Source: Int J Mol Sci. 2024 Jun 19;25(12):6740. doi: 10.3390/ijms25126740 (PMC11204134; doi:10.3390/ijms25126740)
Supplement: Supplementary file 1 [file ijms-25-06740-s001.zip › suppl figure S1.pptx]

## Slide 1
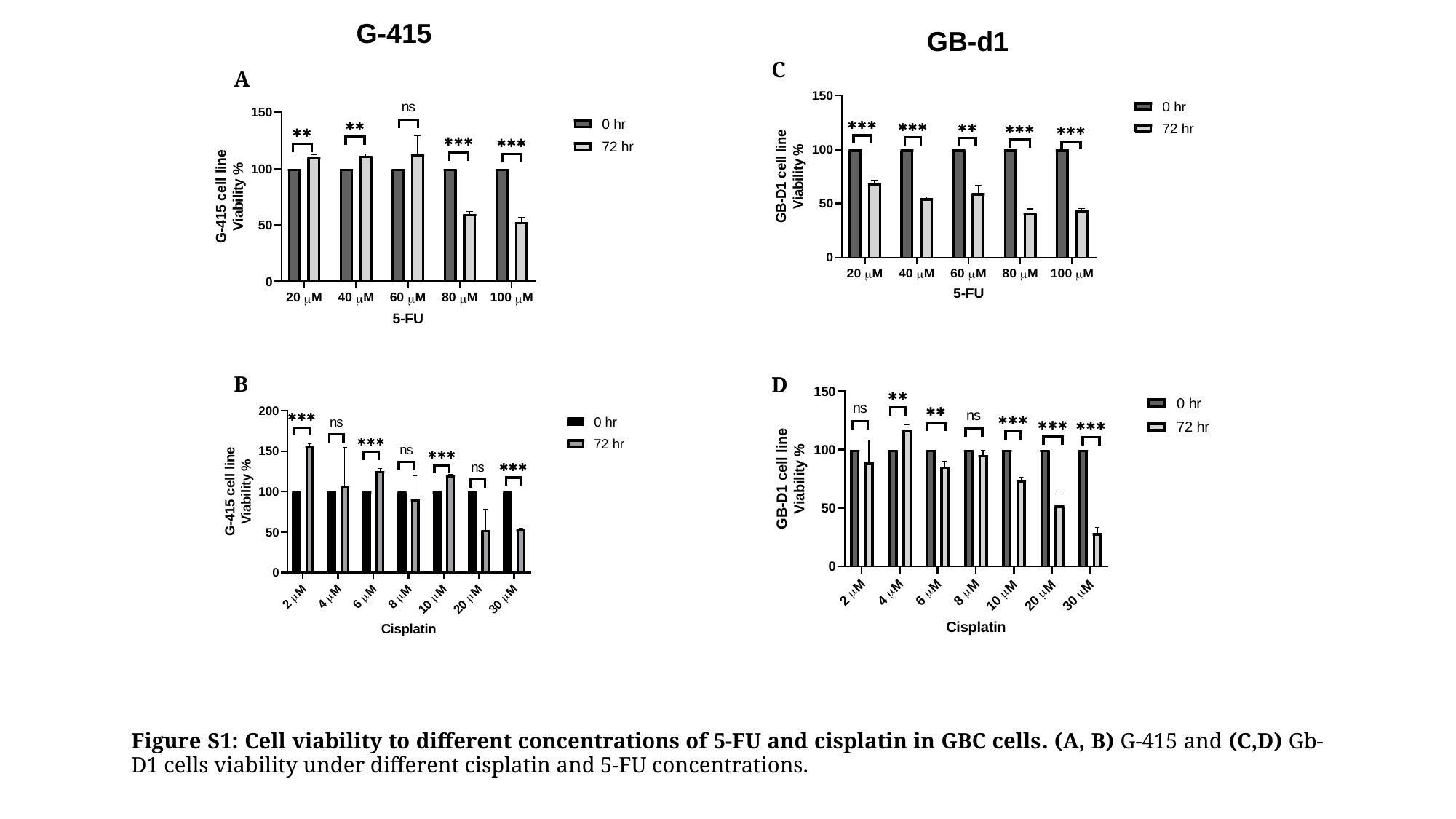

G-415
GB-d1
C
A
B
D
Figure S1: Cell viability to different concentrations of 5-FU and cisplatin in GBC cells. (A, B) G-415 and (C,D) Gb-D1 cells viability under different cisplatin and 5-FU concentrations.

## Slide 2
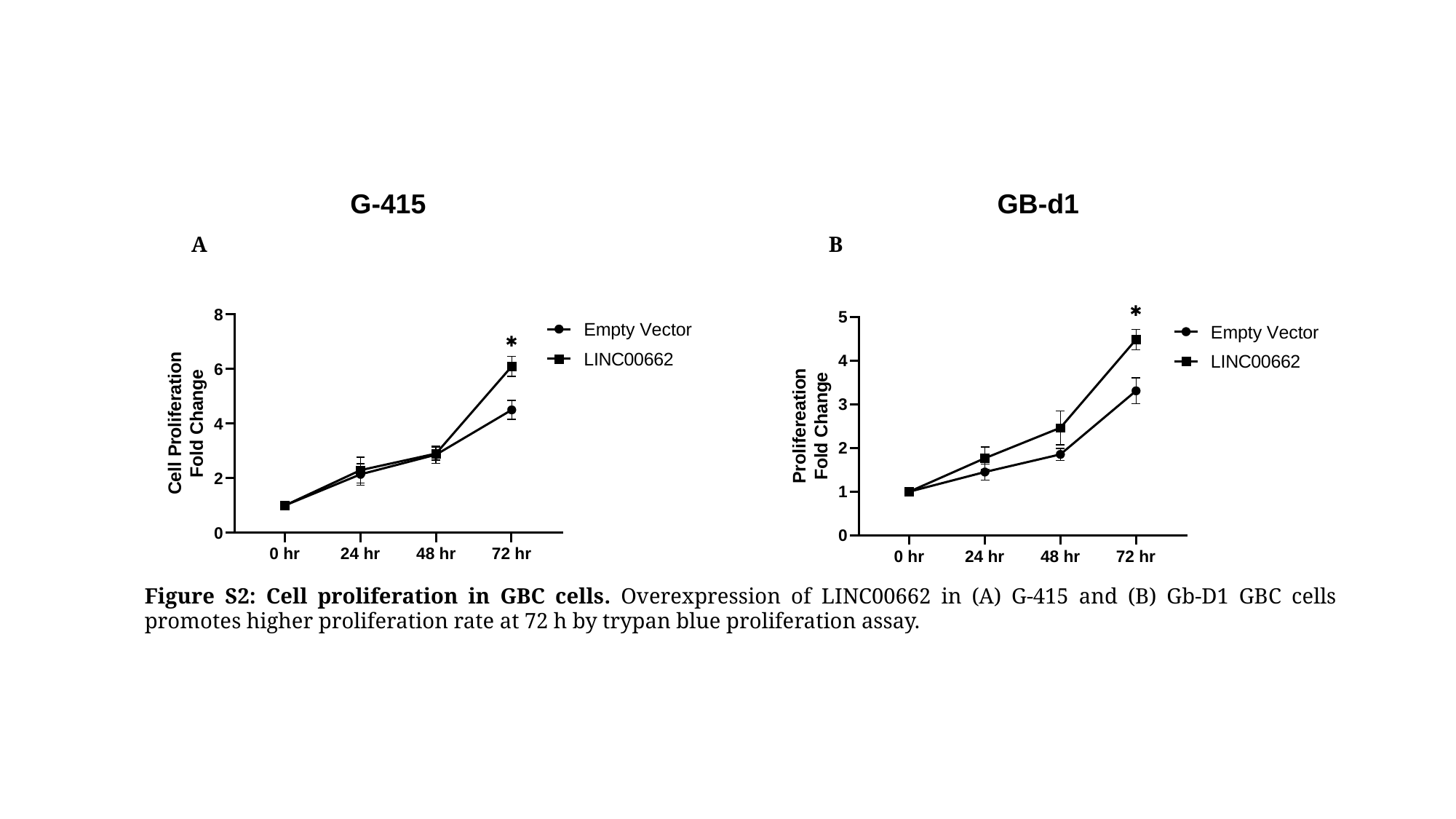

G-415
GB-d1
A
B
Figure S2: Cell proliferation in GBC cells. Overexpression of LINC00662 in (A) G-415 and (B) Gb-D1 GBC cells promotes higher proliferation rate at 72 h by trypan blue proliferation assay.

## Slide 3
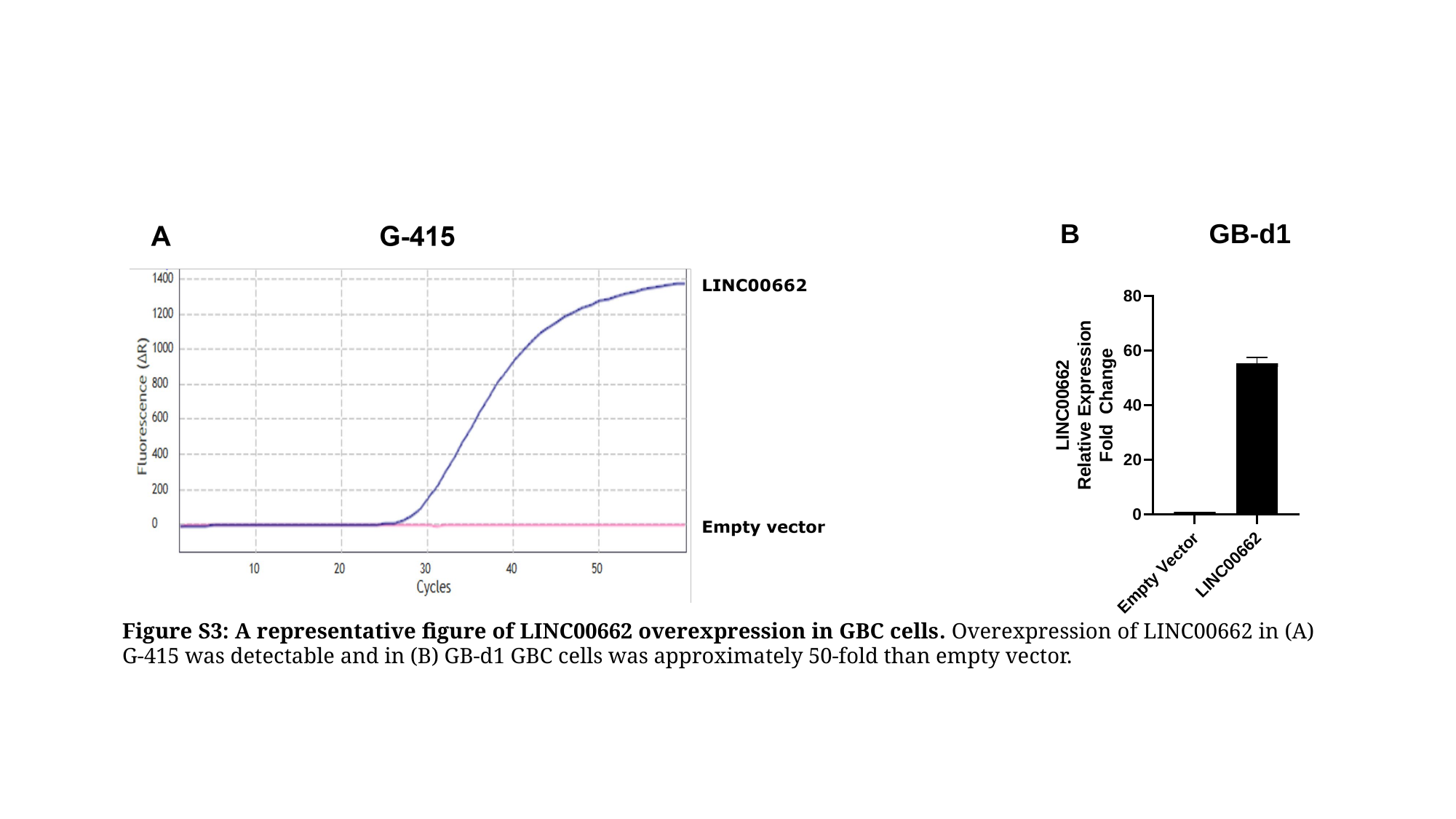

B
GB-d1
Figure S3: A representative figure of LINC00662 overexpression in GBC cells. Overexpression of LINC00662 in (A) G-415 was detectable and in (B) GB-d1 GBC cells was approximately 50-fold than empty vector.
